# Foreword

**Published:** 1988

**Authors:** Martin Mott


### Foreword

### Paediatric Oncology in Bristol

It is now fifteen years since I was appointed to the University Department of Child Health in Bristol with a remit to develop the services for children with cancer. It has taken time, a lot of goodwill and much support to build from those early foundations the multi-disciplinary team which now makes up one of the biggest and most successful children's cancer units in the U.K., but goodwill and support has always been available in abundant measure. Initially, financial help came from many charitable bodies, including the Leukaemia Research Fund, Cancer Research Campaign, Medical Research Council, Malcolm Sargent Fund and others, but in the last decade our major source of support has been the locally based Cancer and Leukaemia in Childhood (CLIC) Trust. Many of the projects reported at this meeting were funded wholly or in part (directly or indirectly) by them, and their continued support is gratefully acknowledged.

At the outset, our first priority was the provision of optimal care for all affected children in the region and the development of support services to help their families during this most difficult ordeal. Because of this primary emphasis on clinical care, our research efforts have predominantly been clinical in nature as can be seen from these proceedings. Research into all aspects of paediatric oncology has been encouraged since a critical review of current practice is an essential requirement for any unit constantly striving to maintain and improve its standards. The success of the clinical unit has enabled us t move onto the next phase of development, exemplifie by the opening of the new CLIC research unit investiga ing the molecular genetics of childhood cancer. It is to b hoped that a look at some fundamental questions abou the biology of these diseases may hold out even brighte therapeutic prospects for the future.

There is one obvious deficiency in this symposium an that is the paucity of research into nursing aspects o paediatric oncology: an explanation of this anomaly i required to avoid a gross injustice to our nursing col leagues.

The standard of care they provide to our patients i exemplary and all of our clinical research would coun for little without their efforts. It is however, a sad fact tha throughout the last decade they have been under stead ly increasing pressures from understaffing and there i little indication that even now their plight has been eithe recognised or acknowledged. Because they rightly pu the clinical care of their patients first, there is little oppor tunity for them to undertake the many important re search projects that they would be in a unique position t accomplish. This situation will continue until the staffing problems are rectified. In the meantime, those o us in the other disciplines that make up the team, ca only record our indebtedness to them.

